# scCapsNet-mask: an updated version of scCapsNet with extended applicability in functional analysis related to scRNA-seq data

**DOI:** 10.1186/s12859-022-05098-8

**Published:** 2022-12-12

**Authors:** Lifei Wang, Rui Nie, Jiang Zhang, Jun Cai

**Affiliations:** 1 Shulan (Hangzhou) Hospital Affiliated to Zhejiang Shuren University Shulan International Medical College, Hangzhou, China; 2 China National Center for Bioinformation, Beijing, 100101 China; 3 Key Laboratory of Genomic and Precision Medicine, Beijing Institute of Genomics, Chinese Academy of Sciences, Beijing, 100101 China; 4 University of Chinese Academy of Sciences, Beijing, 100049 China; 5 School of Systems Science, Beijing Normal University, Beijing, 100875 China

**Keywords:** Capsule network, Interpretability, Mask for sparsity, Non-standard test samples, Functional analysis

## Abstract

**Background:**

With the rapid accumulation of scRNA-seq data, more and more automatic cell type identification methods have been developed, especially those based on deep learning. Although these methods have reached relatively high prediction accuracy, many issues still exist. One is the interpretability. The second is how to deal with the non-standard test samples that are not encountered in the training process.

**Results:**

Here we introduce scCapsNet-mask, an updated version of scCapsNet. The scCapsNet-mask provides a reasonable solution to the issues of interpretability and non-standard test samples. Firstly, the scCapsNet-mask utilizes a mask to ease the task of model interpretation in the original scCapsNet. The results show that scCapsNet-mask could constrain the coupling coefficients, and make a one-to-one correspondence between the primary capsules and type capsules. Secondly, the scCapsNet-mask can process non-standard samples more reasonably. In one example, the scCapsNet-mask was trained on the committed cells, and then tested on less differentiated cells as the non-standard samples. It could not only estimate the lineage bias of less differentiated cells, but also distinguish the development stages more accurately than traditional machine learning models. Therefore, the pseudo-temporal order of cells for each lineage could be established. Following these pseudo-temporal order, lineage specific genes exhibit a gradual increase expression pattern and stem cell associated genes exhibit a gradual decrease expression pattern. In another example, the scCapsNet-mask was trained on scRNA-seq data, and then used to assign cell type in spatial transcriptomics that may contain non-standard sample of doublets. The results show that the scCapsNet-mask not only restored the spatial map but also identified several non-standard samples of doublet.

**Conclusions:**

The scCapsNet-mask offers a suitable solution to the challenge of interpretability and non-standard test samples. By adding a mask, it has the advantages of automatic processing and easy interpretation compared with the original scCapsNet. In addition, the scCapsNet-mask could more accurately reflect the composition of non-standard test samples than traditional machine learning methods. Therefore, it can extend its applicability in functional analysis, such as fate bias prediction in less differentiated cells and cell type assignment in spatial transcriptomics.

**Supplementary Information:**

The online version contains supplementary material available at 10.1186/s12859-022-05098-8.

## Background

Single cell RNA sequencing (scRNA-seq) measures gene expression levels in individual cells and requires diverse computational tools to deal with different computational tasks in the processing pipeline [1–3]. The deep learning model can handle complex data well [4, 5], and has been adopted in a series of necessary steps in the processing pipeline of scRNA-seq data, such as normalization, dimension reduction, and cell type identification [6]. However, the deep learning method lacks interpretability, which is usually operated as a ‘block box’ [7]. Although there have been several attempts to combine biological backgrounds to increase the interpretability of deep learning methods [8–11], this challenge is still not completely solved. Capsule network (CapsNet) is a completely new model for digital recognition, which is different from previous models in mechanism [12], and is expected to be applied to many biological scenarios [13]. Previously, we proposed the single cell capsule network (scCapsNet), a highly interpretable classifier for dealing with scRNA-seq data adopted from CapsNet [14]. Through the internal parameters of the model, scCapsNet could not only classify cell subpopulations with high accuracy, but also reveal the cell type related genes that determine the process of classification. The coupling coefficient is one of these internal parameters, which specifies the relationship between primary capsules and type capsules, and is vital for model interpretation. Due to the random association between primary capsules and type capsules during dynamic routing in scCapsNet, the connections between primary capsules and type capsules are usually dense and redundant, with one-to-many and many-to-one correspondences. These dense and redundant relationships add complexity and difficulty to model interpretation and thus need to be eliminated.

Meanwhile, a challenge for the canonical automatic cell type identification method is to tackle non-standard samples that are not encountered in the training process, such as datasets related to the process of cell development [15]. Cells differentiate from stem cells to terminal committed cells with various cell types. Lineage tracing can track cells across time, linking the cells from a less differentiated state to a more differentiated state [16, 17]. For the machine learning model trained on committed cells (more differentiated), less differentiated cells could be regarded as non-standard samples, which only contain some characteristics of the committed cells. Traditional machine learning methods, such as random forest, will encounter the problem of uniform distribution and hardly distinguish cells according to their differentiated states. As a remedy, an iterative classification strategy is adapted in method FateID for lineage tracing, in order to only deal with the relatively more standard samples in each round. But the iterative nature of FateID makes it vulnerable to trajectory continuity. The lack of intermediate progenitor stages would affect the performance of FateID [18].

The cells with less differentiated states represent one kind of non-standard test sample, the other kind of non-standard test sample occurs in spatial transcriptomics. Human organs are composed of cells with different sources and functions. The spatial position of these cells could provide important information for a better understanding of these organs. Recently, several methods have been developed to infer or measure spatial position [19]. One category is ‘spatial barcoding followed by NGS’, which could capture two-dimensional position information extracted from thin tissue slices [20–22]. However, the resolution of these methods couldn’t be completely achieved at the single cell level. Even for the recently developed near-cellular resolution method Slide-seqV2, multiple cell types in one measurement account for a notable proportion of the whole data [23]. Traditional machine learning models trained on the reference dataset of scRNA-seq have difficulty dealing with these non-standard samples that contain multiple cell types in one measurement. Special methods, such as Robust cell type decomposition (RCTD), have been developed to decompose cell type mixtures in spatial transcriptomes. But it runs relatively slow and requires a lot of computing resources [24].

**Fig. 1:**
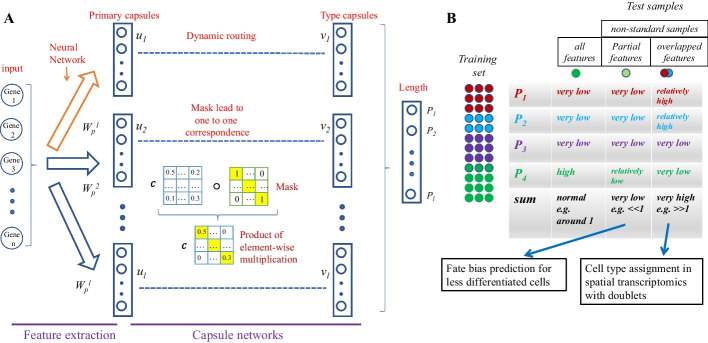
scCapsNet-mask is an updated version of scCapsNet with the capabilities of easier model interpretation and non-standard sample handling. **A** Two-part architecture of scCapsNet-mask. The first part consists of *l* parallel neural networks corresponding to *l* cell types. The primary capsule of vector *u*_*i*_ is the output of the neural network *i*. The subsequent part is a Keras implementation of capsule networks for classification with a mask applied to the parameters of coupling coefficients. The result of the mask is a one-to-one correspondence between primary capsules and type capsules. The length *P*_*j*_ of each type capsule *v*_*j*_ represents the probability of a single cell x belonging to the corresponding cell type.** B** The output of scCapsNet-mask would faithfully reflect the composition of the sample. The scCapsNet-mask was trained on samples with 4 cell types. Each circle represents a cell, and the color of the cell represents the cell type to which it belongs. After model training, if the test sample contains almost all features possessed by one of the training cell types (green circle), the output probability for the corresponding cell type is very high, and the probabilities for the remaining cell types are very low. If the test sample contains few features possessed by one of the training cell types (light green circle), the output probability for the corresponding cell type is relatively low, and the probabilities for the remaining cell types are very low. If the test sample contains features possessed by more than one training cell type (overlapping blue circle and red circle), the output probability for the corresponding cell types is relatively high, and probabilities for the remaining cell types are very low

Here, we introduce single cell capsule network with mask (scCapsNet-mask), an updated version of scCapsNet. Inspired by Sparse Transformers [25], we set the number of primary capsules as the number of type capsules, and constrain the coupling coefficient by adding a mask in the dynamic routing process (Fig. 1A). We first apply scCapsNet-mask to several scRNA-seq datasets [4, 26–28]. The results show that the constrained coupling coefficient is a diagonal square matrix, realizing the one-to-one correspondence between primary capsules and type capsules. Therefore, the scCapsNet-mask reduces the difficulty of model interpretation in the original scCapsNet, and runs automatically without manual inspection. Next, we show that the scCapsNet-mask can handle non-standard samples well. Due to the architecture of CapsNet and the margin loss for classification, the output number for each category is the probability that the input sample belongs to that category (Fig. 1A). After training, if the test sample only contains some features of a specific training category, the output probability falling into that category will be relatively low. Besides, the output probability falling into other categories will be even lower. Thus, the sum of these probabilities for all training categories may be very low (e.g. far less than 1) (Fig. 1B). This could be exemplified in the dataset related cell development. Training on committed cells (more differentiated), the scCapsNet-mask can infer the fate bias of cells with less differentiated states, and then distinguish cells according to their differentiated states. After that, the pseudo-temporal order could be established for each lineage and the results are robust whether the underlined developmental stages are continuous or discontinuous. Otherwise, if the test sample has attributes in two or more training categories, the output probability of these categories is relatively high, and the sum of all probabilities for all training categories is very high (e.g. far more than 1) (Fig. 1B), which was shown when CapsNet tested on the overlapping digits [12]. In biology, this could be exemplified in the dataset related to spatial transcriptomics. Training on scRNA-seq data from the mouse hippocampus [29], the scCapsNet-mask can be used to assign cell types to the spatial transcriptomics of the mouse hippocampus that may contain doublets. It could restore the spatial map, quickly and accurately allocate cell types even for several doublets.

Overall, scCapsNet-mask could provide appropriate solutions to the challenges of interpretability and non-standard test samples. The addition of the mask in scCapsNet-mask eases the task of model interpretation in the original scCapsNet. And the unique type determination mechanism of scCapsNet-mask makes it more suitable for processing non-standard samples, as demonstrated in examples involving cell development and spatial transcriptomics. Therefore, scCapsNet-mask could extend its applicability in downstream functional analysis, which is very important to reveal the biological significance hidden in the data and traditional machine learning models can rarely do this [6].

## Results

### scCapsNet-mask: an updated version of the interpretable classifier scCapsNet

In the experiments, the datasets of mRBC profiled by the Drop-Seq, the datasets of human kidney and the dataset of hPBMC profiled by the 10X Genomics were used to test the prediction accuracy of scCapsNet-mask. The training set and test set are generated by randomly shuffle-splitting the datasets at a ratio of 9:1. The scCapsNet-mask is run several times with different shuffle-splits to assess its prediction accuracy. The scCapsNet-mask performs well on classification tasks. The average prediction accuracy is around 99%, 97%, 90% for mRBC, hPBMC and the kidney dataset, respectively, comparable to scCapsNet and other machine learning methods (Additional file 1: Fig S1) [14].

**Fig. 2:**
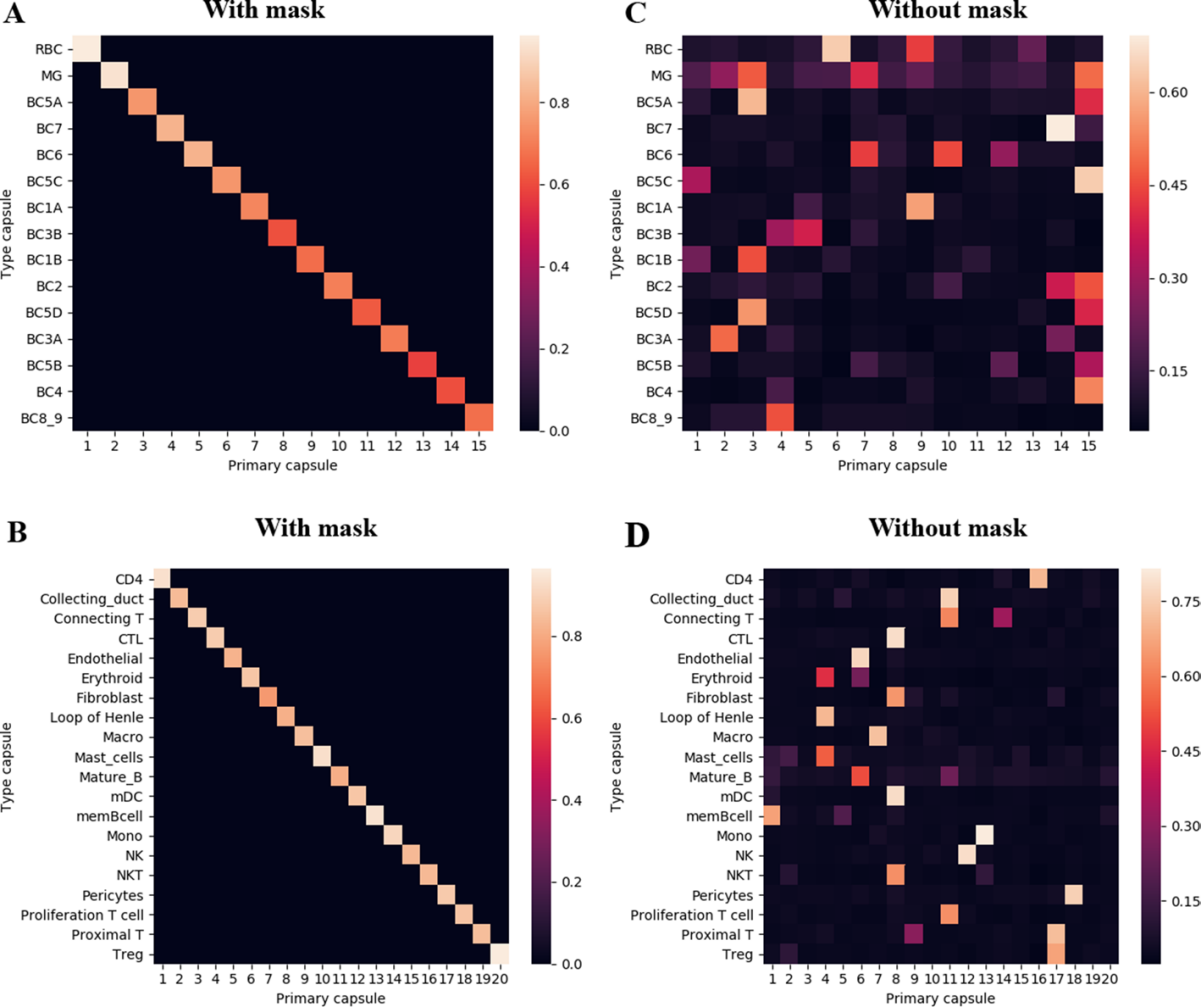
The overall average coupling coefficients show that mask leads to the one-to-one correspondence between primary capsules and type capsules. **A, B** The overall average coupling coefficients (overall heatmap) generated by scCapsNet-mask, which contains the effective type capsule row in each of the average coupling coefficients in the mRBC **A** or human kidney **B** dataset. The overall average coupling coefficients show that applying the mask leads to a one-to-one correspondence between primary capsules and type capsules. **C, D **The overall average coupling coefficient generated by scCapsNet without mask, which contains the effective type capsule row in each of the average coupling coefficients in the mRBC **C** or human kidney **D** dataset. The overall average coupling coefficients show that the lack of mask will lead to one-to-many and many-to-one correspondences between primary capsules and type capsules

Both scCapsNet and scCapsNet-mask are composed of an input layer (gene), a primary capsule layer, and a type capsule layer (cell type). As described by scCapsNet, the genes in the first layer can be associated with the cell type in the last layer, that is, the interpretability of the scCapsNet and scCapsNet-mask. In these models, the average coupling coefficients relates the primary capsule to the type capsule (cell types), and the weight matrix further relate genes to cell types [14] (Fig. 1A). However, due to the random association between primary capsules and type capsules during dynamic routing, the average coupling coefficient generated by the original scCapsNet is complex. The addition of the mask in the scCapsNet-mask is supposed to constrain the coupling coefficients. The results generated by the scCapsNet-mask show that there is only one most active element in each average coupling coefficient (Additional file 1: Figs. S2, S3). Furthermore, the overall average coupling coefficients for all cell types demonstrate that only the on-diagonal elements are active, and each primary capsule is only associated with one type capsule and vice versa (Fig. 2A, B). Otherwise, in the results generated by the original scCapsNet, there will be many-to-one and one-to-many correspondences between the primary capsules and type capsules (Fig. 2C, D, Additional file 1: Figs. S4, S5). These results undoubtedly justify our proposal for the effect of the mask, that is, the mask would constrain the weight distribution of the coupling coefficients and concentrate the weights on the on-diagonal elements. After applying the mask, there is a one-to-one correspondence between primary capsules and type capsules, unlike the many-to-one or one-to-many correspondence between primary capsules and type capsules existing in the original scCapsNet. Subsequently, the model could automatically find the relationship between the primary capsules and type capsules, without needing manual inspection to indicate which primary capsule responds to the recognition of which cell types.

Furthermore, this one-to-one correspondence between primary capsules and type capsules generated by mask also makes the association between genes and primary capsules (cell type) easier. As described in the previous version of scCapsNet, the internal weight matrix in the network connecting the inputs with each primary capsule would further associate genes from inputs with each cell type. The Principal Component Analysis (PCA) performed on the column vectors of the internal weight matrix for particular cell type would roughly order the genes according to their importance in the classification of that cell type [14]. But due to the many-to-one or one-to-many correspondence between primary capsules and type capsules, the ordering of the genes may follow the other Principal Component (e.g. Principal Component 2 for Megakaryocytes in [14]) rather than Principal Component 1. For scCapsNet-mask, the ordering of the genes usually following the Principal Component 1 (PC1) according to empirical observation and the fact of the one-to-one correspondence, makes it easy to select the specific group of genes responsible for the recognition of a particular cell type (Additional file 1: Fig S6). For example, in the mRBC dataset, the selection of cell-type associated genes is all along the Principal Component 1 (PC1). And those cell-type related genes selected by scCapsNet-mask model contain many bio-markers such as *Prkca*, *Apoe*, *Sox6*, *Igfn1*, *Lect1*, *Slitrk5*, *Pcdh17*, *Nnat*, *Wls*, *Syt2*, *Lrrtm1*, *Erbb4*, *Chrm2*, *Col11a1*, and *Serpini1* for RBC, Müller glia (MG) and several types of cone bipolar cells, respectively [14] (Additional file 1: Fig S6B, colored stars). Overall, the above results demonstrate that the scCapsNet-mask retains the merits of the original scCapsNet and eases the complexity and difficulty of model interpretation.

### scCapsNet-mask could specify the fate bias and development stage of non-standard samples with less differentiated states, after training on committed cells

**Fig. 3:**
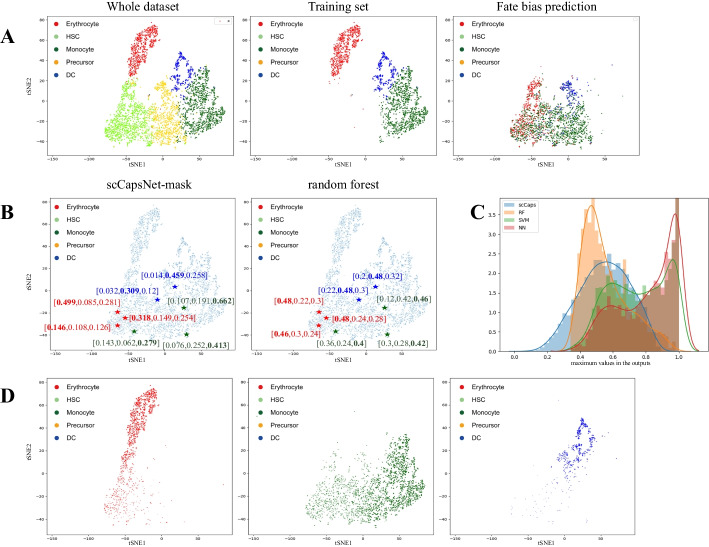
The scCapsNet-mask could estimate the fate bias and development stage of the cells with less differentiated states after training on committed cells. **A** Left: the tSNE plot of early human hematopoietic stem cells (HSC) differentiation in the bone marrow [30]. Middle: the scCapsNet-mask trained on committed cells (erythrocytes, monocytes, and dendritic cells). Right: the fate bias of non-standard samples (precursor and HSC) are estimated by the trained scCapsNet-mask model. **B** Comparison of probabilities output by scCapsNet-mask and random forest. The star represents the HSC or progenitor cells. The colors of the star represent the lineage to which the HSC or progenitor cells belong to, as predicted by scCapsNet-mask or random forest. Left: the probabilities output by scCapsNet-mask, the sum of these probabilities is not strict to one. The maximum values of output range from low values to high values, which could be used to distinguish the development stage. Right: the probabilities output by random forest, the sum of these probabilities is strict to one. The maximum values of output squeeze in a narrow range, which could not be used to distinguish the development stage. **C** The distributions of the maximum values in the output for all non-standard test samples (less differentiated cells). The distributions generated by support vector machine (SVM) and neural networks (NN) are skewed to high values (near 1) and the distribution of random forest (RF) is skewed to middle values (near 0.5). In contrast, the maximum values in the outputs of the scCapsNet-mask (scCaps) are distributed more evenly, ranging from low values to high values.** D** The pseudo-temporal order of cells for each cell lineage according to the maximum values in the output of scCapsNet-mask. The depth of the color represents the degree of development, while the dark color corresponds to the more differentiated state and the light color corresponds to the less differentiated state. Left: erythrocytes. Middle: monocytes. Right: dendritic cells

In the experiment, the dataset of human hematopoietic stem cells differentiation in bone marrow was used, which consisted of monocytes, dendritic cells (DCs), erythrocytes, human hematopoietic stem cells (HSCs) and precursors (Fig. 3A, Left) [30]. The scCapsNet-mask and several machine learning methods (random forest, support vector machine, neural networks) are trained on the committed cell populations (monocytes, DCs, and erythrocytes) at first (Fig. 3A, Middle), and then tested on HSCs and precursors that are regarded as non-standard samples from the perspective of committed cells. The outputs of the models indicate the probability of a given precursor or HSC belonging to each corresponding lineage (committed cell populations) (Fig. 3B). For outputs of the scCapsNet-mask, several less differentiated cells (non-standard) have low predicted probabilities for every lineage ([0.146, 0.108, 0.126], [0.143, 0.062, 0.279], [0.032, 0.309, 0.12]). The sum of the output is even lower than 0.5 (0.38, 0.484, 0.461), which is far less than 1 (Fig. 3B, Left). In contrast, the sum of the output for the random forest is equal to 1.

The lineage with the maximum value in the output would be selected as the fate bias of the precursor or HSC (Fig. 3B). Therefore, the precursors and HSCs would be allocated into one of the lineages (Fig. 3A, Right). At the same time, this maximum value in the output of scCapsNet-mask could be used as a measure for the development stage of the non-standard sample (HSCs and precursors), which is difficult to achieve by other methods such as random forest (Fig. 3B, C). For example, the comparison experiment demonstrates 3 precursors and HSCs which are predicted to differentiate to erythrocytes (Fig. 3B, red star), with the maximum values of 0.146, 0.318, 0.499 inferred by scCapsNet-mask (Fig. 3B, Left) and the maximum values of 0.46, 0.48, 0.48 inferred by random forest (Fig. 3B, Right). These maximum values inferred by scCapsNet-mask could distinguish the development stage, while the maximum value inferred by random forest couldn't. The same phenomenon was observed in the other two lineages (Fig. 3B). In order to understand the situation more comprehensively, the distributions of the maximum values in the output for all non-standard test samples (less differentiated cells) are also plotted (Fig. 3C). The results show that the maximum values in the outputs of the support vector machine and neural networks are skewed to high values (near 1) and the maximum values in the outputs of the random forest are skewed to middle values (near 0.5). None of the maximum values generated by traditional machine learning methods reach low values (close to 0). In contrast, the maximum values in the outputs of the scCapsNet-mask are distributed more evenly, ranging from low values to high values. So, the cells could be more evenly ordered by their maximum values in output of scCapsNet-mask, as a higher value corresponds to a more differential state and a lower value corresponds to a less differential state. Therefore, the pseudo-temporal order of cells in each lineage could be inferred, as they move forward along differentiation trajectories from stem cells to committed cell populations (Fig. 3D).

**Fig. 4:**
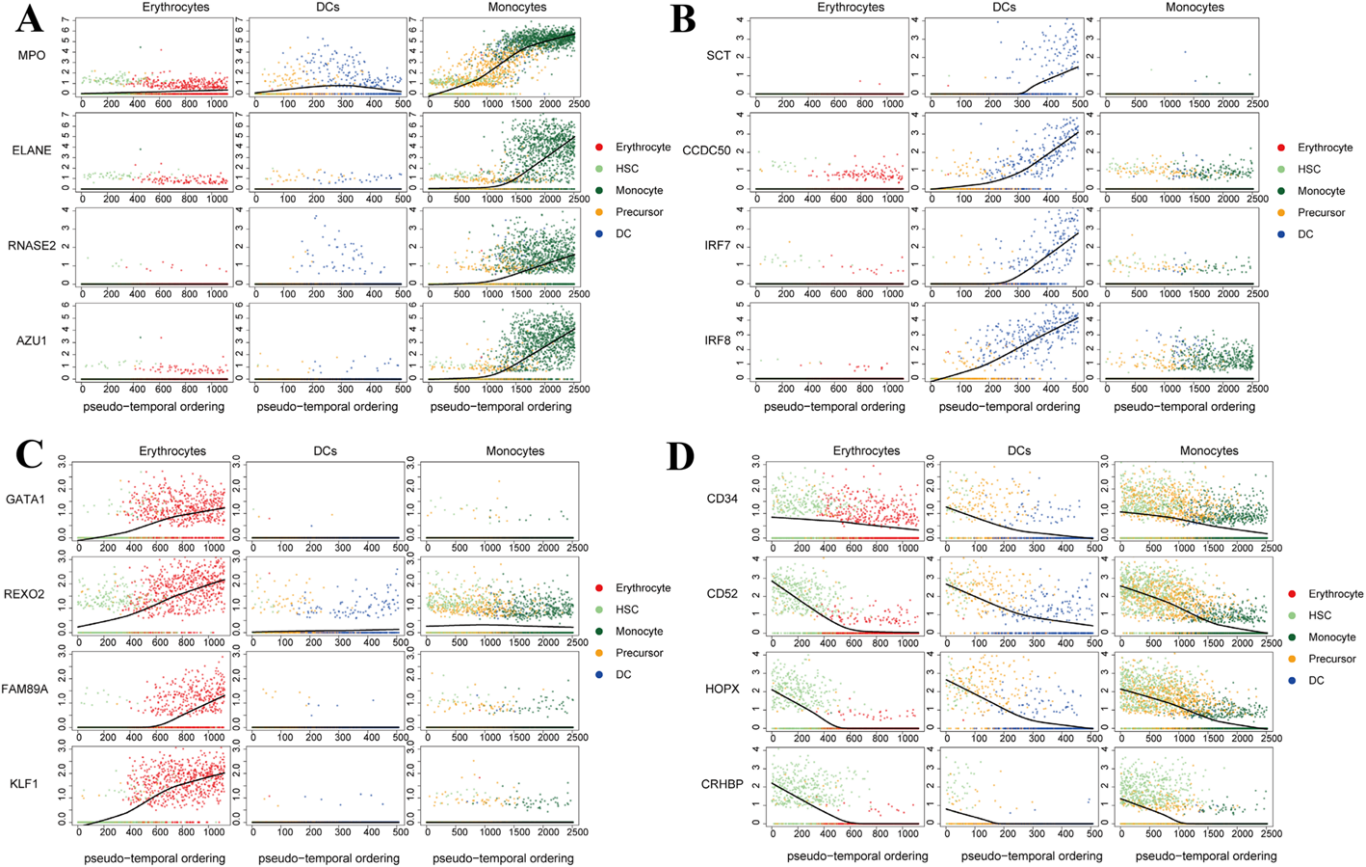
The pseudo-temporal order of each cell lineage reveals a gradual increase in expression of lineage specific genes for each cell lineage and a gradual decrease in the expression of HSC associated genes in all cell lineages. **A** Expression profiles of 4 monocytes-specific genes (*MPO*, *ELANE*, *RNASE2*, *AZU1*) in the process of cell differentiation of 3 lineages. **B** Expression profiles of 4 DCs-specific genes (*SCT*, *CCDC50*, *IRF7*, *IRF8*) in the process of cell differentiation of 3 lineages.** C** Expression profiles of 4 erythrocytes-specific genes (*GATA1*, *REXO2*, *FAM89A*, *KLF1*) in the process of cell differentiation of 3 lineages.** D** Expression profiles of 4 HSC-specific genes (*CD34*, *CD52*, *HOPX*, *CRHBP*) in the process of cell differentiation of 3 lineages.

Following pseudo-temporal orders generated by scCapsNet-mask, the genes belonged to lineage-specific genes with a gradual increase in expression, and genes related to HSCs with a gradual decrease in expression (Fig. 4). For example, the genes related to monocytes (*MPO*, *ELANE*, *RNASE2,* and *AZU1*) [18, 31–33] are subjected to gradual increase of the expression in pseudo-temporal order of monocyte development. In contrast, the expression levels of these genes were almost not changed in the pseudo-temporal orders of other lineages (Fig. 4A). Pseudo-temporal ordering of dendritic cells revealed a gradual increase in the expression of genes related to dendritic cells, such as the marker gene *IRF8* [34], the regulators of adaptive immunity *IRF7* [35], the highly expressed gene *CCDC50* in the Plasmacytoid dendritic cell (pDC) [36], and the induced immature DC chemotaxis gene *SCT* during DC development [37] (Fig. 4B). Pseudo-temporal ordering of erythrocytes also showed similar trends. The erythrocytes related genes (*GATA1*, *KLF1*, *REXO2,* and *FAM89A*) [18, 38, 39] gradually increased in their expression in pseudo-temporal order of erythrocytes development but did not change in the pseudo-temporal ordering of dendritic cells and monocytes (Fig. 4C). In addition, the genes related to HSC (*CD34*, *CD52*, *CRHBP,* and *HOPX*) [40–43] are gradually decreased along the pseudo-temporal ordering of all three lineages (Fig. 4D). Taken together, these results suggest that the pseudo-temporal orders reproduced well the changes in expression along the developmental trajectory of several lineage-specific and HSC-related genes. This can only be achieved by the fact that scCaspNet-mask accurately captures the developmental stage of non-standard samples.

Furthermore, the fate bias predicted by one-step methods such as scCapsNet-mask is more robust, it’s the same whether the intermediate progenitors are missing. In contrast, for the iterative method FateID, its performance would be dramatically affected when the intermediate precursor stage is missing. The absence of these intermediate precursors would alter the fate bias prediction of the HSC, where the fate bias toward the DC lineage almost disappears (Additional file 1: Fig S7).

### scCapsNet-mask could identify non-standard samples of doublets in spatial transcriptomics, after training on the scRNA-seq data

**Fig. 5:**
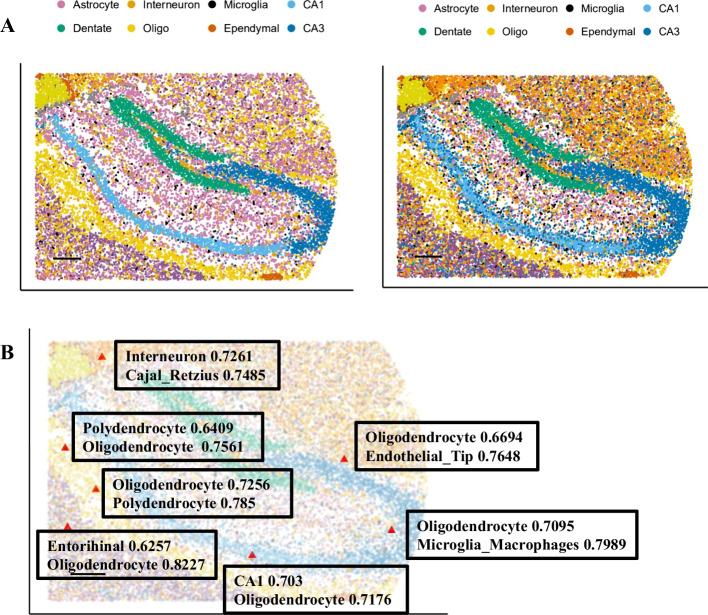
The scCapsNet-mask assigns cell types in the spatial transcriptomics of the hippocampus and identifies several doublets. **A** The spatial map of predicted cell types in the hippocampus generated by RCTD [24] (Left) and scCapsNet-mask (Right). Of 17 cell types, the 8 most commonly appear in the legend.** B** The locations with two relatively high prediction probabilities by scCapsNet-mask are marked as red triangles in the spatial map of the hippocampus.

The dataset of scRNA-seq or single nucleus RNA sequencing (snRNA-seq) could be used as reference for supervised learning methods to assign cell types in spatial transcriptomics, which may contain non-standard samples of cells mixtures [23, 24]. In the experiment, the scCapsNet-mask model was first trained on scRNA-seq data of 17 cell types in the mouse hippocampus [24], and then tested on the Slide-seqV2 data of the mouse hippocampus. The results showed that the spatial map of predicted cell types generated by scCapsNet-mask model is consistent with that generated by RCTD and the anatomical structure of the mouse hippocampus [24], with less time and computing resources (Fig. 5A). For most cell types (such as CA1, CA3, Choroid, Dentate, Ependymal, Interneuron, Neuron.Slc17a6, Oligodendrocyte, and so on), scCapsNet-mask assigns them to the appropriate spatial position (Additional file 1: Fig S8–S10). There are some differences between scCapsNet-mask and RCTD in cell type assignment in Astrocyte, Cajal_Retzius, Entorhinal, and Polydendrocyte (Additional file 1: Fig S11). Furthermore, scCapsNet-mask identifies several doublets in spatial transcriptomics, which has a relatively high probability for two cell types (Fig. 5B). These relatively high probabilities of the two cell types (0.6257 for Entorhinal, 0.8227 for Oligodendrocyte) indicate that there may be a mixture of two cell types at this position, which could not be achieved by traditional machine learning methods with the restriction that the sum of outputs is equal to one.

## Discussion

Here, we have developed an updated version of the interpretable model scCapsNet, aiming to alleviate the difficulty and complexity of model interpretation caused by the one-to-many and many-to-one correspondence between primary capsules and type capsules in the original version. In the process of dynamic routing, the masking mechanism is introduced to limit the weight distribution of the coupling coefficients, so as to achieve the bijective mapping (one-to-one correspondence) between the primary capsules and type capsules. We named this updated version interpretable model scCapsNet-mask. After testing on several scRNA-seq datasets, the results shows that the bijective mapping (one-to-one correspondence) between primary capsules and type capsules is realized in scCapsNet-mask. And multiple cell type related genes could be revealed as original scCapsNet.

In addition to interpretable cell type identification, we also demonstrated the capability of scCapsNet-mask to properly handle the non-standard sample. Due to its unique category determining process (architecture of CapsNet and the margin loss for classification), the output number for each category predicted by scCapsNet-mask represents the absolute probability of that category, not the normalized probability. Therefore, the test sample that has never been encountered in the training process would have a low probability for all training categories; and the test sample from a mixture with multiple training categories would have a high probability for corresponding training categories. These characteristics of scCapsNet-mask are utilized to handle non-standard samples, which could reveal the composition of the non-standard samples more accurately.

In one example, the scCapsNet-mask and other machine learning methods were trained on committed cells and tested on cells with less differentiated states. Compared with the traditional machine learning methods, the outputs of the scCapsNet-mask could better infer the fate bias and distinguish the differentiated stages of the less differentiated cells, due to their more even distribution and wider range. According to the results of scCapsNet-mask, the pseudo-temporal order could be established for each lineage. And these pseudo-temporal orders could correctly reproduce the gradual increase of lineage specific genes for each lineage and the gradual decrease of stemness marker genes for all lineages, which further proves the validity of our method.

In another example, the scCapsNet-mask was trained on scRNA-seq datasets of the hippocampus and tested on corresponding spatial transcriptomics generated by Slide-seqV2, which may contain the mixture of cell types as the non-standard samples. The scCapsNet-mask could identify the mixture of cell types by assigning two high probabilities, while traditional machine learning methods couldn't.

As shown in the above two examples, according to the composition of the test samples, scCapsNet-mask would output low probabilities for all training categories or several high probabilities (> 0.5) for several training categories. The capability of anomaly (non-standard sample) detection and the interpretable nature of scCapsNet-mask could further extend its applicability in functional analysis. One possible application area is research involving tumors. The scCapsNet-mask could be trained on normal cell populations and tested on tumor cells. The trained scCapsNet-mask model may measure the similarity between tumor cells and normal cells, thus inferring the source of the tumor cells with heterogeneous composition and different origins. Therefore, the scCapsNet-mask may contribute to the research on cancer progression and metastasis.

## Conclusions

The scCapsNet-mask is an interpretable classifier with the capability of handling non-standard samples. It uses a mask to realize the one-to-one correspondence between the primary capsules and type capsules, which greatly reduces the difficulty of model interpretation in the original scCapsNet. Furthermore, the output of scCapsNet-mask would faithfully reflect the composition of the sample. When the sample is quite different from the reference, all items in the output are low; when the sample contains a mixture of cell types, many items in the outputs are relatively high. This feature makes the scCapsNet-mask more suitable for processing non-standard samples, and then extends its applicability in functional analysis, such as fate bias prediction in less differentiated cells and cell type assignment in spatial transcriptomics.

## Methods

### The RNA-seq datasets and data preprocessing

We evaluated the values of our method for single-cell transcriptome analysis using Drop-Seq single cell data of mouse retinal bipolar cells (mRBC) [4, 27], 10X Genomics single cell data of human peripheral blood mononuclear cells (hPBMC) [4, 26], and 10X Genomics single cell data of the tissue of kidney [28]. The transcriptome profiles included ~ 20,000 single mRBCs with average ~ 13,000 genes from 15 subcellular groups and ~ 12,000 single hPBMCs with average ~ 3300 genes from 8 subcellular groups, and ~ 35,000 cells with average ~ 6000 highly variable genes selected by using Conos 1.4.9 from 20 cell types [44]. All data were log-transformed before use.

The 10X single-cell RNA-seq datasets of early human hematopoietic stem cells (HSC) differentiation in bone marrow were downloaded from Setty et al. [45]. The analysis was performed according to Zhou et al. [30]. Briefly, we used the Scanpy package to normalize with total Unique Molecular Identifier(UMI) count per cell [46], select 1000 highly-variable genes, and renormalize after filtering for 4142 cells. All data were log-transformed before use.

We downloaded Slide-seqV2 and scRNA-seq datasets in the hippocampus [24]. The analysis was performed according to Cable et al. [24]. Briefly, the single cell RNA-seq datasets in the hippocampus include 17 cell types, whose cell numbers are above 25 and subsampled 1000 in each cell type. Then we selected the differentially expressed gene in each cell type compared to all cell types, the cutoff was set log2Foldchange > 1.25 and a minimum average expression above 0.00015. After that, we acquired ~ 5000 genes for evaluating the methods.

### scCapsNet-mask model

The scCapsNet-mask is based on scCapsNet. A detailed description of scCapsNet architecture was previously described [14]. There are two major differences between scCapsNet and scCapsNet-mask. First, there are *l* neural networks in the scCapsNet-mask corresponding to *l* cell types in the dataset, and each neural network uses Rectified Linear Unit (ReLU) or tanh as the activation function. On the contrary, in scCapsNet, the number of the neural networks is designated as a hyperparameter.

1 $$u_{i} = ReLU\left( {W_{p}^{i} x} \right) i \in \left[ {1,2 \ldots ,l} \right]$$

Secondly, in “dynamic routing” process, scCapsNet-mask has an additional step after calculating the coupling coefficient. The coupling coefficients need to multiply element-wise with a mask matrix, which is an identity matrix, with on-diagonal elements all being one and the off-diagonal elements all being zero. This operation concentrates the weights in the on-diagonal elements and ignores the off-diagonal elements of the coupling coefficient.

2 $$c = {\text{c}}\circ m$$

The implementation of the model is demonstrated by the following pseudocode.
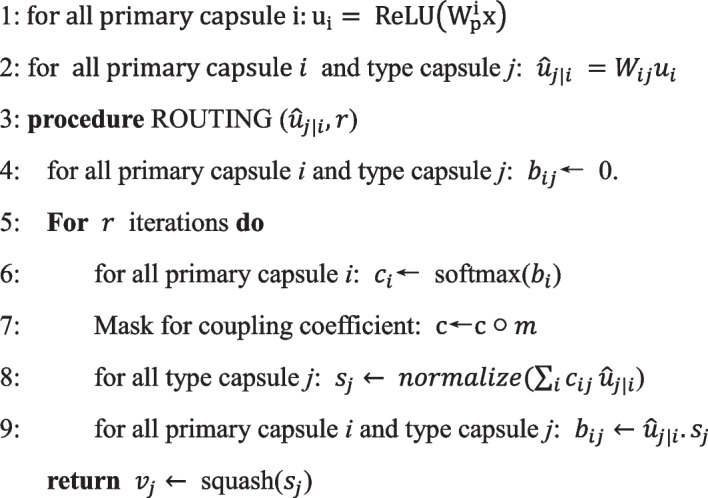


The implementation of scCapsNet-mask can be found in https://github.com/wanglf19/scCapsNet_mask.

### Margin loss for type classification

As in the capsule networks, the probability that an input sample belongs to a certain cell type is represented by the length of the corresponding type capsule (vector) in scCapsNet-mask. And only when the input sample belongs to a certain cell type, the length of the corresponding type capsule is long. The separate margin loss is used to train each type capsule [12, 14].

3 $$L_{k} = T_{k} max\left( {0,m^{ + } - ||v_{k} ||} \right)^{2} + \lambda \left( {1 - T_{k} } \right) \, max\left( {0, \, \left| {\left| {v_{k} } \right|} \right| - m^{ - } } \right)^{2}$$

where *T*_*k*_ = 1 if the input sample belongs to that cell type according to the training label and *m*^+^  = 0.9 and *m*^*−*^  = 0.1. The λ is down-weighting of the loss and set to 0.25 in scCapsNet-mask. The total loss is the sum of the losses of all type capsules.

**Average Coupling coefficients** As described in scCapsNet[14] and multiCapsNet [47], the coupling coefficients connect the primary capsules and type capsules. Every input sample (single cell) will produce its own coupling coefficients. The average coupling coefficients for each cell type are shown below:

4 $$c_{ij}^{type average} = \frac{{\mathop \sum \nolimits_{type} c_{ij}^{type} }}{{\mathop \sum \nolimits_{type} 1}}$$

The average coupling coefficient matrix for each cell type contains an effective type capsule row. The overall average coupling coefficient matrix (overall heatmap) is composed of the effective type capsule rows from all cell types ($c_{i1}^{type 1 average} , c_{i2}^{type 2 average} , c_{i3}^{type 3 average}$…) [47].

### Implementation and hyperparameters

We implement the scCapsNet-mask in the environment of Python 3.6, conda 4.4.10, keras 2.2.4, and tensorflow 1.11.0 on a notebook computer equipped with intel(R) Core(TM) i5-7300HQ CPU@2.50 GHz and 8 GB RAM. The user could establish the environment through code “conda create -n sccaps python = 3.6 keras = 2.2.4 tensorflow = 1.11.0”.

The ‘randoms’ and ‘test_size’ are the hyperparameters of the method sklearn.model_selection.train_test_split that used in scCapsNet-mask, in order to randomly split dataset into training and test subsets. The ‘randoms’ controls the shuffling of the data before dataset splitting, and the ‘test_size’ controls the proportion of the dataset to include in the test subset. The ‘dim_capsule’ specifies the dimension of the primary and type capsule. The default value is 16 for original CapsNet. We recommend setting this value to roughly twice the number of cell types. The hyperparameters ‘lr’, ‘batch_size’ and ‘epoachs’ relate to the model training. The ‘lr’ is the learning rate for adam optimizers. When the loss is ‘NaN’ during the training, please reduce the learning rate and retrain the model. The model parameters are updated in each batch, and the ‘batch_size’ defines the number of samples in the batch. The ‘epochs’ specify the number of times the model will train for an entire training dataset. The ‘epochs’ can be adjusted according to the loss of training. The hyperparameters ‘pc_slice’ and ‘threshold’ are associated with selecting the specific group of genes responsible for the recognition of a particular cell type. The ‘pc_slice’ specifies the fineness of division along the PC direction. The ‘threshold’ is the threshold for the prediction accuracy of the specific cell type, so that a dotted line is set when the prediction accuracy of the specific cell type is just below the threshold.

## Methods for comparison

### Traditional machine learning methods

A neural network with a softmax activation function was implemented in Keras. The support vector machine and random forest were implemented with the Python package ‘scikit-learn’.

### FateID

We applied the FateID from the R package FateID 0.2.1 to infer cell fate bias in multipotent progenitors from the scRNA-seq dataset with default parameters [18].

### RCTD

We applied the RCTD from the R package spacexr 2.0.0 to decomposition of cell type mixtures in spatial transcriptomics with default parameters [24].

### Estimate the fate-bias of cells with less differentiated states and order cells in pseudo-temporal order

As FateID, we first train scCapsNet-mask model on the committed cell populations (Monocytes, dendritic cells (DCs), and erythrocytes), and use the trained model to measure the similarity between the cells with less differentiated states (HSCs and Precursors) and the committed cell population for each lineage. Then the lineage with the highest similarity is considered as the fate bias for that HSC or precursor. The pseudo-temporal order of cells in each lineage is established through the absolute value of the fate bias (maximum values in the outputs) for each cell. Then, the expression of the lineage specific genes for each lineage and stemness marker genes are examined.

## Supplementary Information

**Additional file 1**.**Figure S1**. The performance for single-cell type recognition in the scCapsNet-mask is comparable to that of scCapsNet and other machine learning methods. The training accuracies and testing accuracies of scCapsNet-mask (mask), scCapsNet, neural network, random forest and support vector machine (SVM) on the human kidney dataset are plotted. The box-and-whisker plots drawn by boxplot from R show the training and testing accuracies in nine replicates of each method.** Figure S2**. The average coupling coefficients show that the mask leads to the one-to-one correspondence between primary capsules and type capsules in the mRBC dataset. The average coupling coefficients (heatmaps) generated by scCapsNet-mask for the mRBC dataset with the cell types listed above. The row represents type capsules and the column represents primary capsules in each heatmap. These heatmaps show that applying the mask leads to a one-to-one correspondence between primary capsules and type capsules.** Figure S3**. The average coupling coefficients show that the mask leads to the one-to-one correspondence between primary capsules and type capsules in the human kidney dataset. The average coupling coefficients (heatmaps) generated by scCapsNet-mask for the human kidney dataset with cell types listed above. The row represents type capsules and the column represents primary capsules in each heatmap. These heatmaps show that applying the mask leads to a one-to-one correspondence between primary capsules and type capsules.** Figure S4**. The average coupling coefficients show that lacking the mask leads to the complex correspondences between primary capsules and type capsules in the mRBC dataset. The average coupling coefficients (heatmaps) generated by scCapsNet-mask for the mRBC dataset with the cell types listed above. The row represents type capsules and the column represents primary capsules in each heatmap. These heatmaps show that lacking mask leads to the complex correspondence between primary capsules and type capsules.** Figure S5**. The average coupling coefficients show that lacking the mask leads to the complex correspondences between primary capsules and type capsules in the human kidney dataset. The average coupling coefficients (heatmaps) generated by scCapsNet-mask for the human kidney dataset with cell types listed above. The row represents type capsules and the column represents primary capsules in each heatmap. These heatmaps show that lacking the mask leads to the complex correspondence between primary capsules and type capsules.** Figure S6**. The scCapsNet-mask could identify cell type associated genes along Principal Component 1 in mRBC dataset.** A** Each colored line represents the prediction accuracy for cell types along principal component in B. The y axis represents prediction accuracy, the x axis represents principal component in B and the dotted lines are corresponding to the chosen genes of blue dots in B. In the dotted lines, recognition accuracy degrades close to 0 for corresponding cell type listed above whereas other cell types are not significantly affected.** B** The plot depicts the two-dimensional PCA on the weight matrix for the primary capsule. Each dot represents a gene and the blue dots are chosen as cell type related genes (dotted line associated genes in A). The colored stars with labels represent the marker genes for each cell type (*Prkca, Apoe, Sox6, Igfn1, Lect1, Slitrk5, Pcdh17, Nnat, Wls, Syt2, Lrrtm1, Erbb4, Chrm2, Col11a1, Serpini1*).** Figure S7**. The fate bias inferred by FateID is less robust against the missing intermediate progenitor stages.** A** Left: The FateID trained on erythroid cell, monocytes, and dendritic cells. Middle: the fate bias of precursor and HSC estimated by FateID. Right: the fate bias of HSCs estimated by FateID when precursors are absent.** B** The composition of fate bias is dramatically changed for HSC population after the intermediate progenitor population is missing.** Figure S8**. Predicted spatial localization of cell types by RCTD and scCapsNet-mask in the Slide-seqV2 hippocampus. Left: CA1, CA3, Choroid, Dentate in RCTD. Right: CA1, CA3, Choroid, Dentate in scCapsNet-mask.** Figure S9**. Predicted spatial localization of cell types by RCTD and scCapsNet-mask in the Slide-seqV2 hippocampus. Left: Entorhinal, Interneuron, Neuron.Slc17a6, Oligodendrocyte in RCTD. Right: Entorhinal, Interneuron, Neuron.Slc17a6, Oligodendrocyte in scCapsNet-mask.** Figure S10**. Predicted spatial localization of cell types by RCTD and scCapsNet-mask in the Slide-seqV2 hippocampus. Left: Microglia_Macrophages, Mural, Neurogenesis, Endothelial_Stalk, Endothelial_Tip in RCTD. Right: Microglia_Macrophages, Mural, Neurogenesis, Endothelial_Stalk, Endothelial_Tip in scCapsNet-mask.** Figure S11**. Predicted spatial localization of cell types by RCTD and scCapsNet-mask in the Slide-seqV2 hippocampus. Left: Astrocyte, Cajal_Retzius, Entorhinal, Polydendrocyte in RCTD. Right: Astrocyte, Cajal_Retzius, Entorhinal, Polydendrocyte in scCapsNet-mask.

## Data Availability

scCapsNet-mask model is open source, and available for download from https://github.com/wanglf19/scCapsNet_mask. It is implemented in python 3.6.

## Abbreviations

scCapsNet: Single cell capsule network
scRNA-seq: Single cell RNA sequencing
snRNA-seq: Single nucleus RNA sequencing
CapsNet: Capsule network
RCTD: Robust cell type decomposition
mRBC: Mouse retinal bipolar cells
hPBMC: Human peripheral blood mononuclear cells
NGS: Next-generation sequencing
PCA: Principal component analysis
PC1: Principal component 1
MG: Müller glia
pDC: Plasmacytoid dendritic cell
DC: Dendritic cell
HSC: Human hematopoietic stem cell
UMI: Unique molecular identifier
ReLU: Rectified linear unit
