# Supplementary material for: scCapsNet-mask: an updated version of scCapsNet with extended applicability in functional analysis related to scRNA-seq data
Source: BMC Bioinformatics. 2022 Dec 12;23:539. doi: 10.1186/s12859-022-05098-8 (PMC9743530; doi:10.1186/s12859-022-05098-8)
Supplement: Supplementary file 1 — Additional file 1.Figure S1. The performance for single-cell type recognition in the scCapsNet-mask is comparable to that of scCapsNet and other machine learning methods. The training accuracies and testing accuracies of scCapsNet-mask (mask), scCapsNet, neural network, random forest and support vector machine (SVM) on the human kidney dataset are plotted. The box-and-whisker plots drawn by boxplot from R show the training and testing accuracies in nine replicates of each method. Figure S2. The average coupling coefficients show that the mask leads to the one-to-one correspondence between primary capsules and type capsules in the mRBC dataset. The average coupling coefficients (heatmaps) generated by scCapsNet-mask for the mRBC dataset with the cell types listed above. The row represents type capsules and the column represents primary capsules in each heatmap. These heatmaps show that applying the mask leads to a one-to-one correspondence between primary capsules and type capsules. Figure S3. The average coupling coefficients show that the mask leads to the one-to-one correspondence between primary capsules and type capsules in the human kidney dataset. The average coupling coefficients (heatmaps) generated by scCapsNet-mask for the human kidney dataset with cell types listed above. The row represents type capsules and the column represents primary capsules in each heatmap. These heatmaps show that applying the mask leads to a one-to-one correspondence between primary capsules and type capsules. Figure S4. The average coupling coefficients show that lacking the mask leads to the complex correspondences between primary capsules and type capsules in the mRBC dataset. The average coupling coefficients (heatmaps) generated by scCapsNet-mask for the mRBC dataset with the cell types listed above. The row represents type capsules and the column represents primary capsules in each heatmap. These heatmaps show that lacking mask leads to the complex correspondence between pri [file 12859_2022_5098_MOESM1_ESM.pdf]

Fig S1

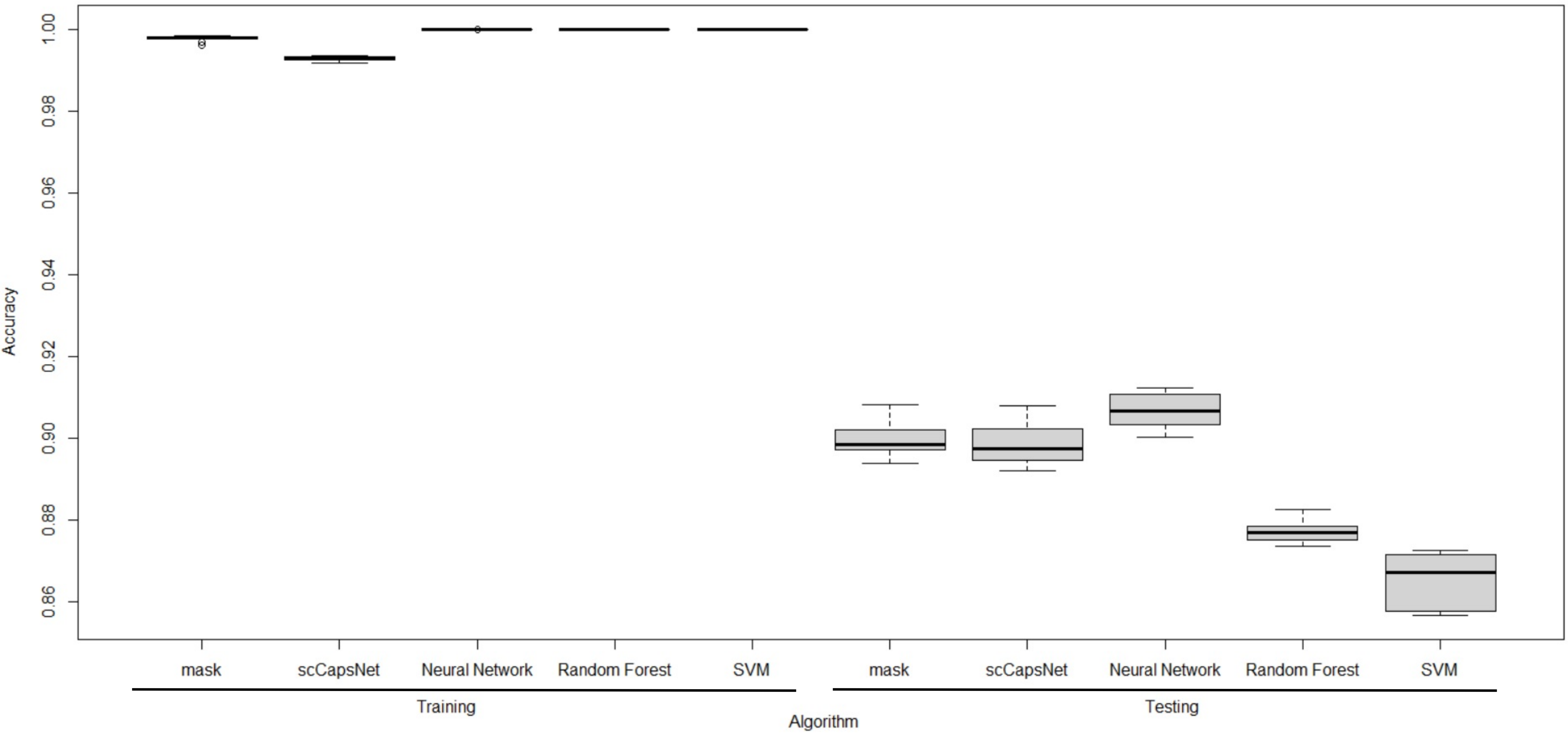
Accuracy
1.00
0.98
0.96
0.94
0.92
0.90
0.88
0.86
mask
scCapsNet
Neural Network
Random Forest
SVM
mask
scCapsNet
Neural Network
Random Forest
SVM
Training
Testing
Algorithm

Fig S2

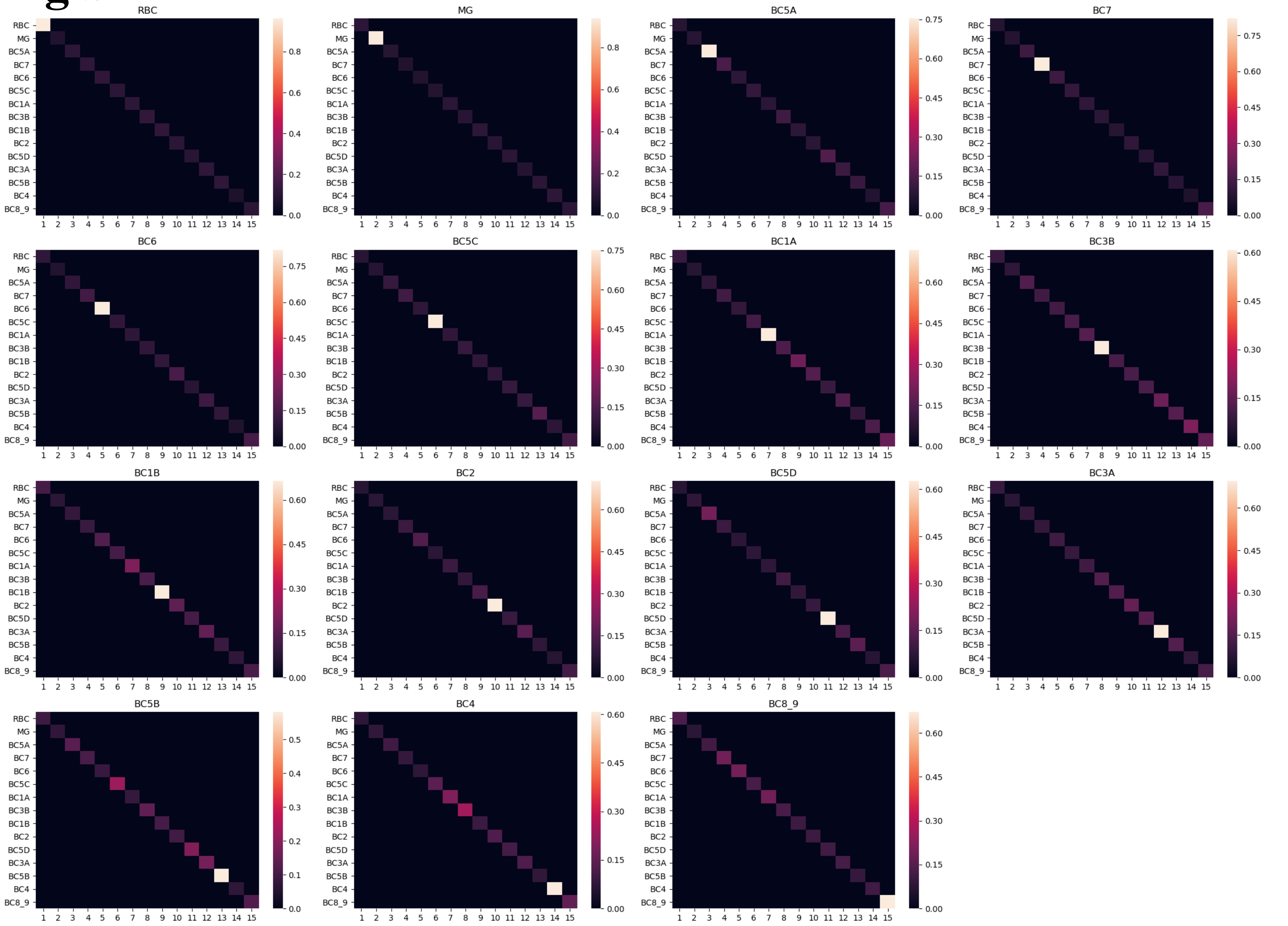
RBC
MG
BC5A
BC7
BC6
BC5C
BC1A
BC3B
BC1B
BC2
BC5D
BC3A
BC5B
BC4
BC8_9
1 2 3 4 5 6 7 8 9 10 11 12 13 14 15

Fig S3

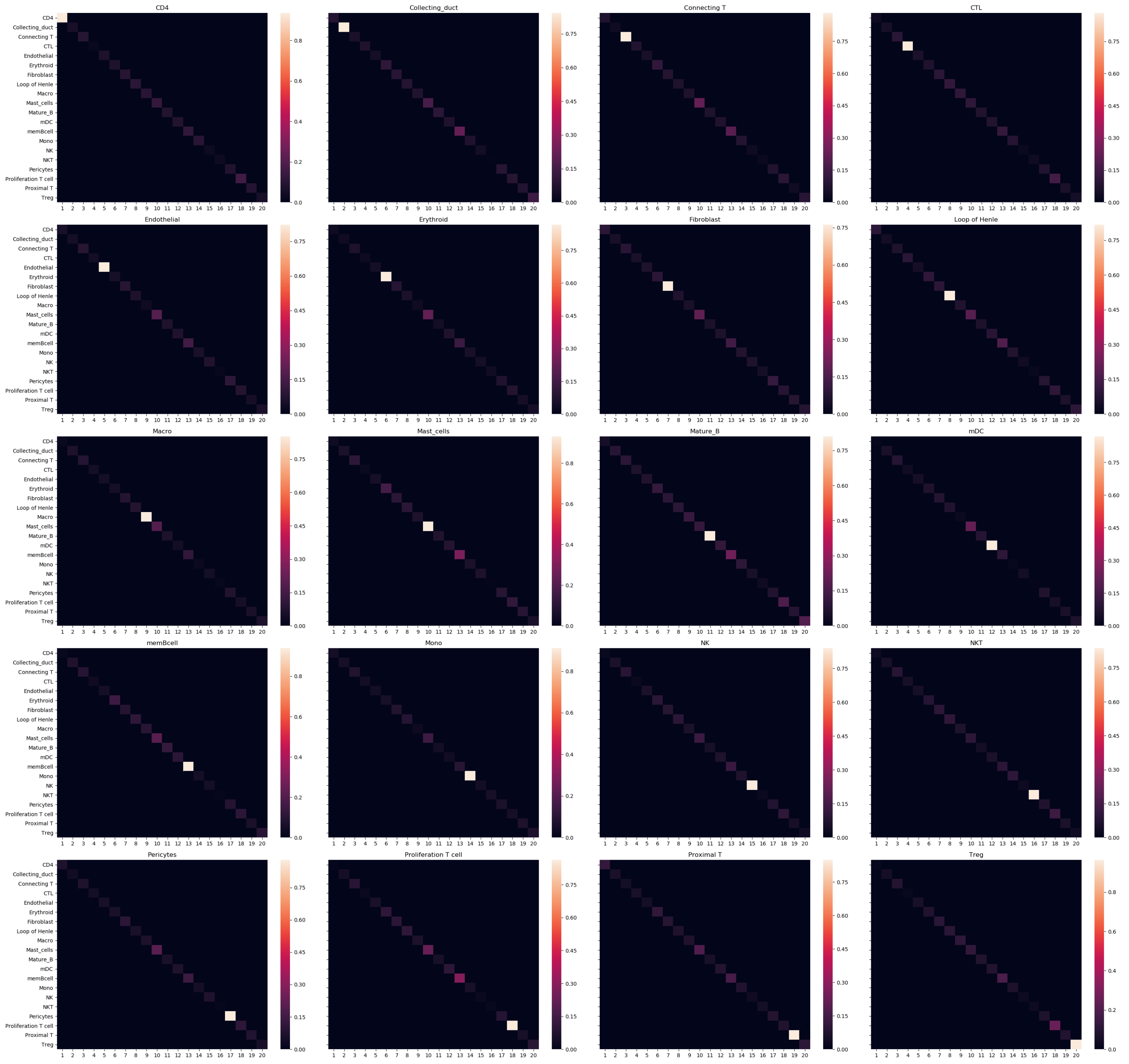
CD4
Collecting_duct
Connecting T
CTL
Endothelial
Erythroid
Fibroblast
Loop of Henle
Macro
Mast_cells
Mature_B
mDC
memBcell
Mono
NK
NKT
Pericytes
Proliferation T cell
Proximal T
Treg
1 2 3 4 5 6 7 8 9 10 11 12 13 14 15 16 17 18 19 20

Fig S4

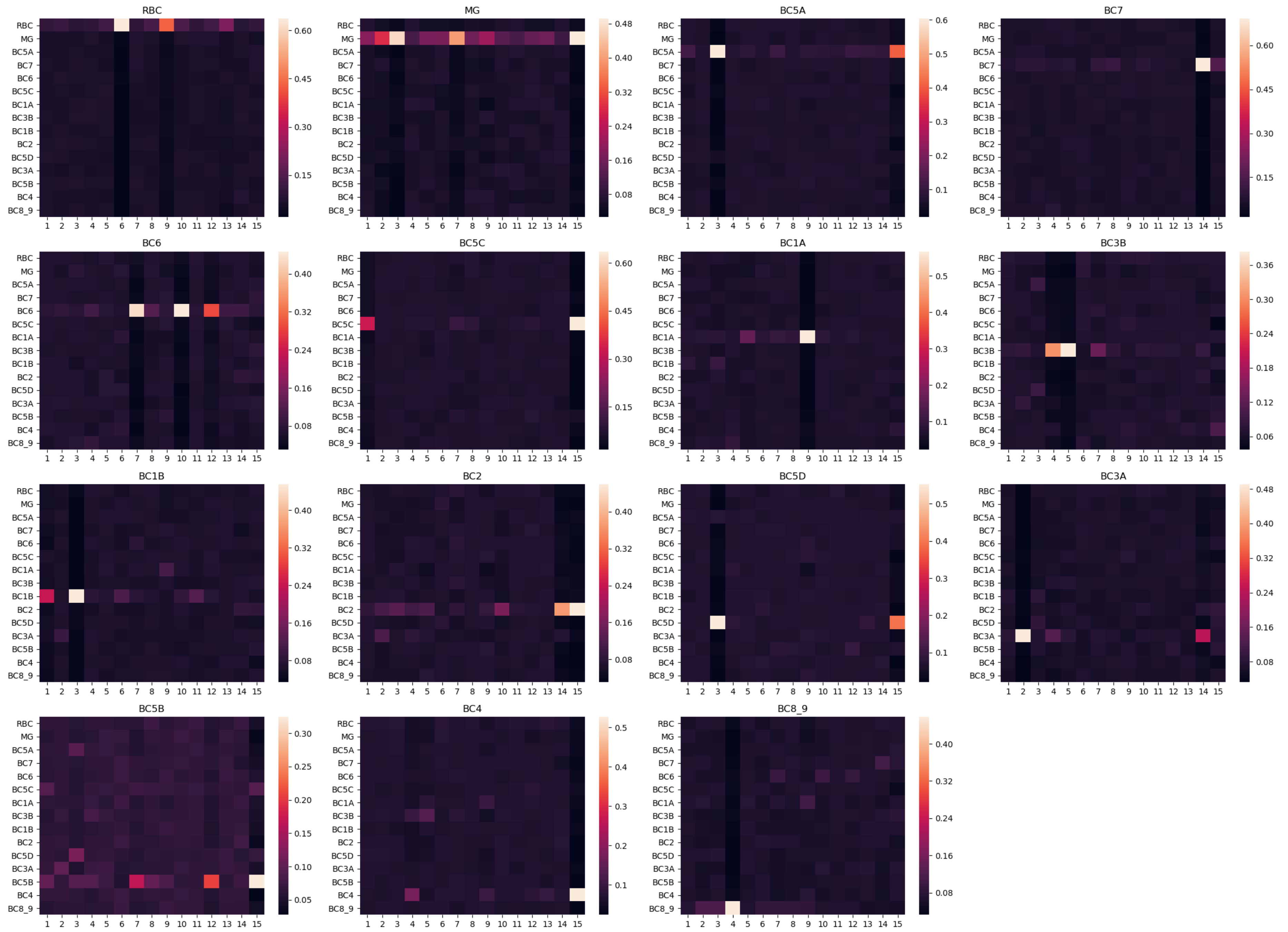
RBC
MG
BC5A
BC7
BC6
BC5C
BC1A
BC3B
BC1B
BC2
BC5D
BC3A
BC5B
BC4
BC8_9
1 2 3 4 5 6 7 8 9 10 11 12 13 14 15

Fig S5

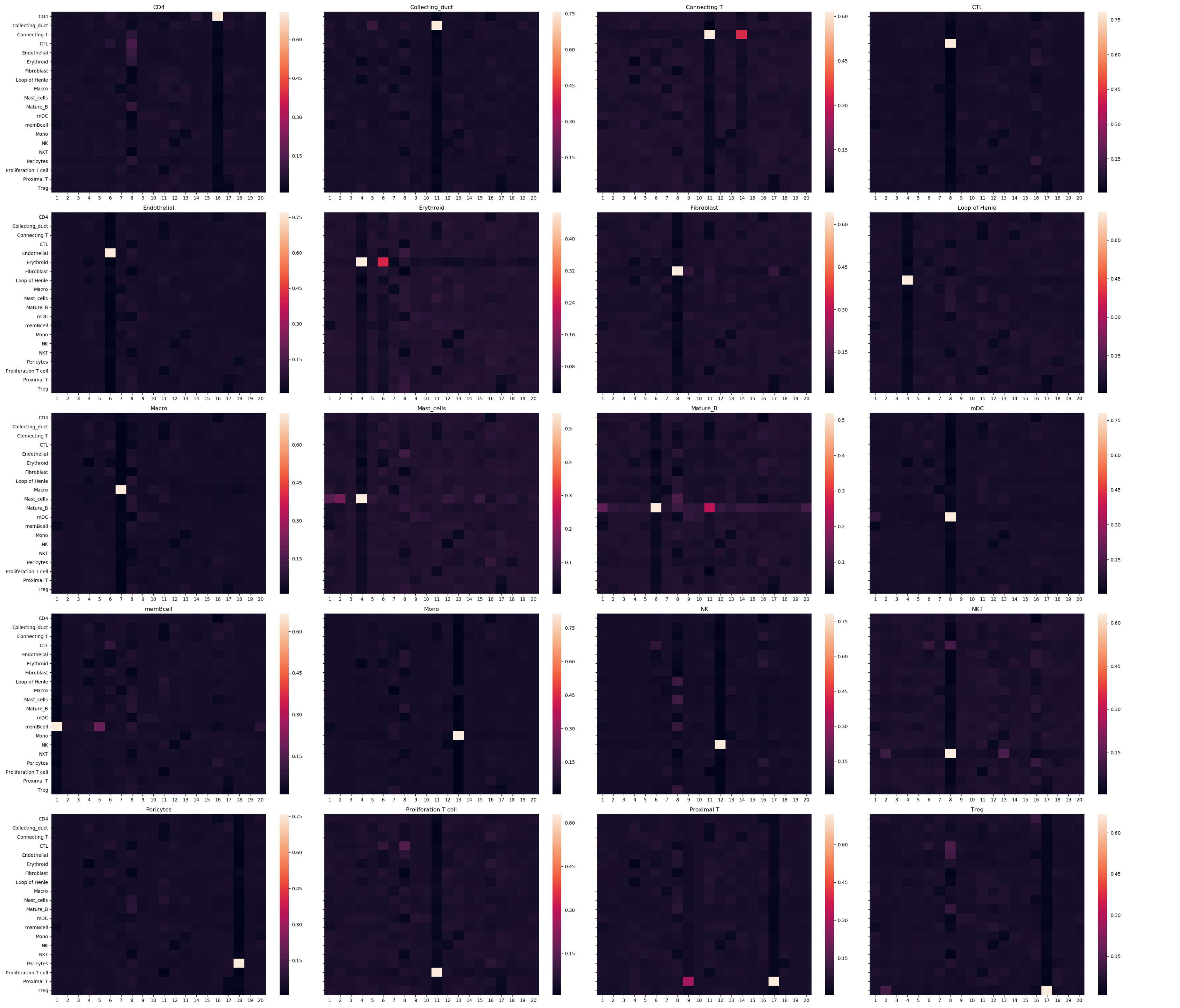

CD4
Collecting_duct
Connecting T
CTL
Endothelial
Erythroid
Fibroblast
Loop of Henle
Macro
Mast_cells
Mature_B
mDC
memBcell
Mono
NK
NKT
Pericytes
Proliferation T cell
Proximal T
Treg

Fig S6

A

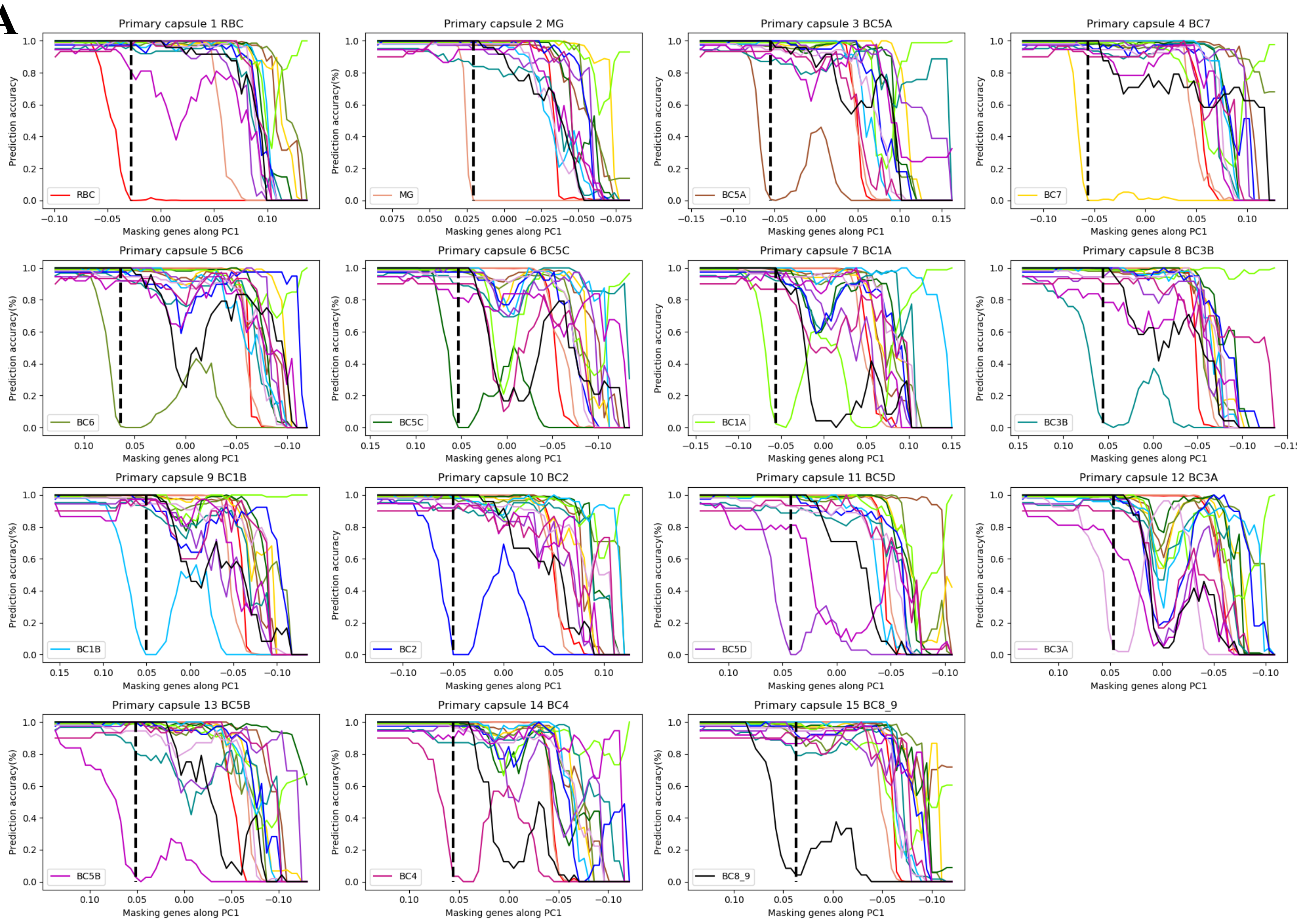

Primary capsule 1 RBC
Primary capsule 2 MG
Primary capsule 3 BC5A
Primary capsule 4 BC7
Primary capsule 5 BC6
Primary capsule 6 BC5C
Primary capsule 7 BC1A
Primary capsule 8 BC3B
Primary capsule 9 BC1B
Primary capsule 10 BC2
Primary capsule 11 BC5D
Primary capsule 12 BC3A
Primary capsule 13 BC5B
Primary capsule 14 BC4
Primary capsule 15 BC8_9
Prediction accuracy
Prediction accuracy(%)
Masking genes along PC1
RBC
MG
BC5A
BC7
BC6
BC5C
BC1A
BC3B
BC1B
BC2
BC5D
BC3A
BC5B
BC4
BC8_9

B

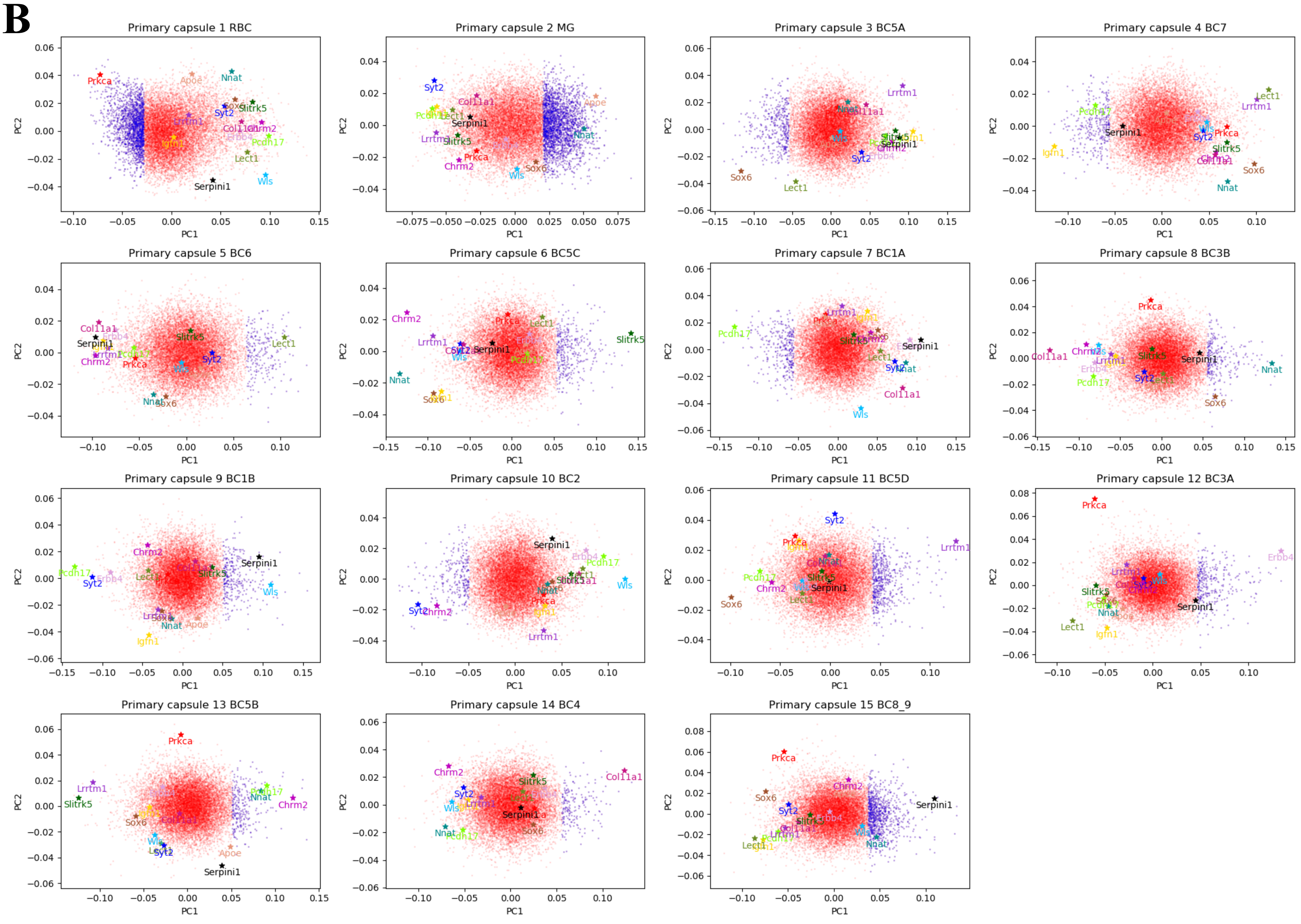

Primary capsule 1 RBC
Primary capsule 2 MG
Primary capsule 3 BC5A
Primary capsule 4 BC7
Primary capsule 5 BC6
Primary capsule 6 BC5C
Primary capsule 7 BC1A
Primary capsule 8 BC3B
Primary capsule 9 BC1B
Primary capsule 10 BC2
Primary capsule 11 BC5D
Primary capsule 12 BC3A
Primary capsule 13 BC5B
Primary capsule 14 BC4
Primary capsule 15 BC8_9
PC1
PC2
Prkca
Apoe
Nnat
Syt2
Sox6
Slitrk5
Chrm2
Pcdh17
Lect1
Wls
Serpini1
Col11a1
Lrrtm1
Igfn1
Erbb4

Fig S7

A

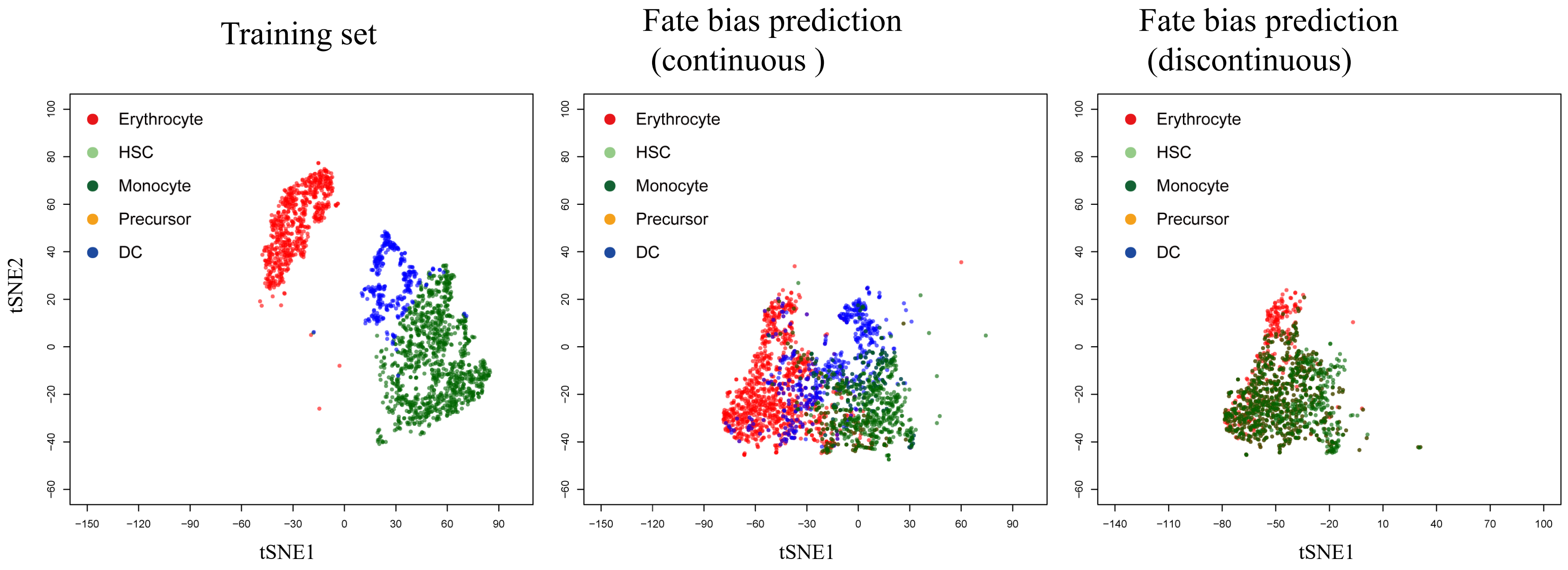
Training set
Fate bias prediction (continuous)
Fate bias prediction (discontinuous)
Erythrocyte
HSC
Monocyte
Precursor
DC
tSNE2
tSNE1

B

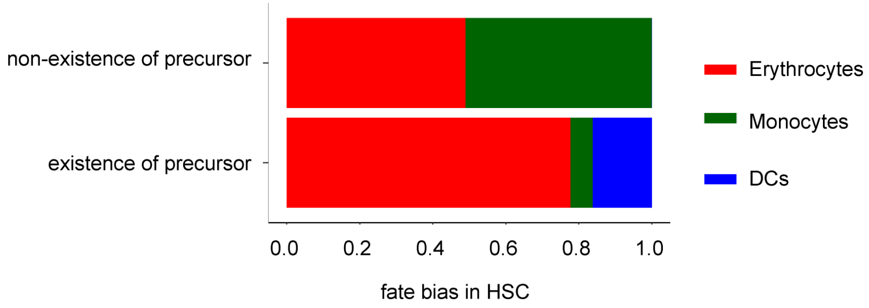
non-existence of precursor
existence of precursor
Erythrocytes
Monocytes
DCs
0.0
0.2
0.4
0.6
0.8
1.0
fate bias in HSC

Fig S8

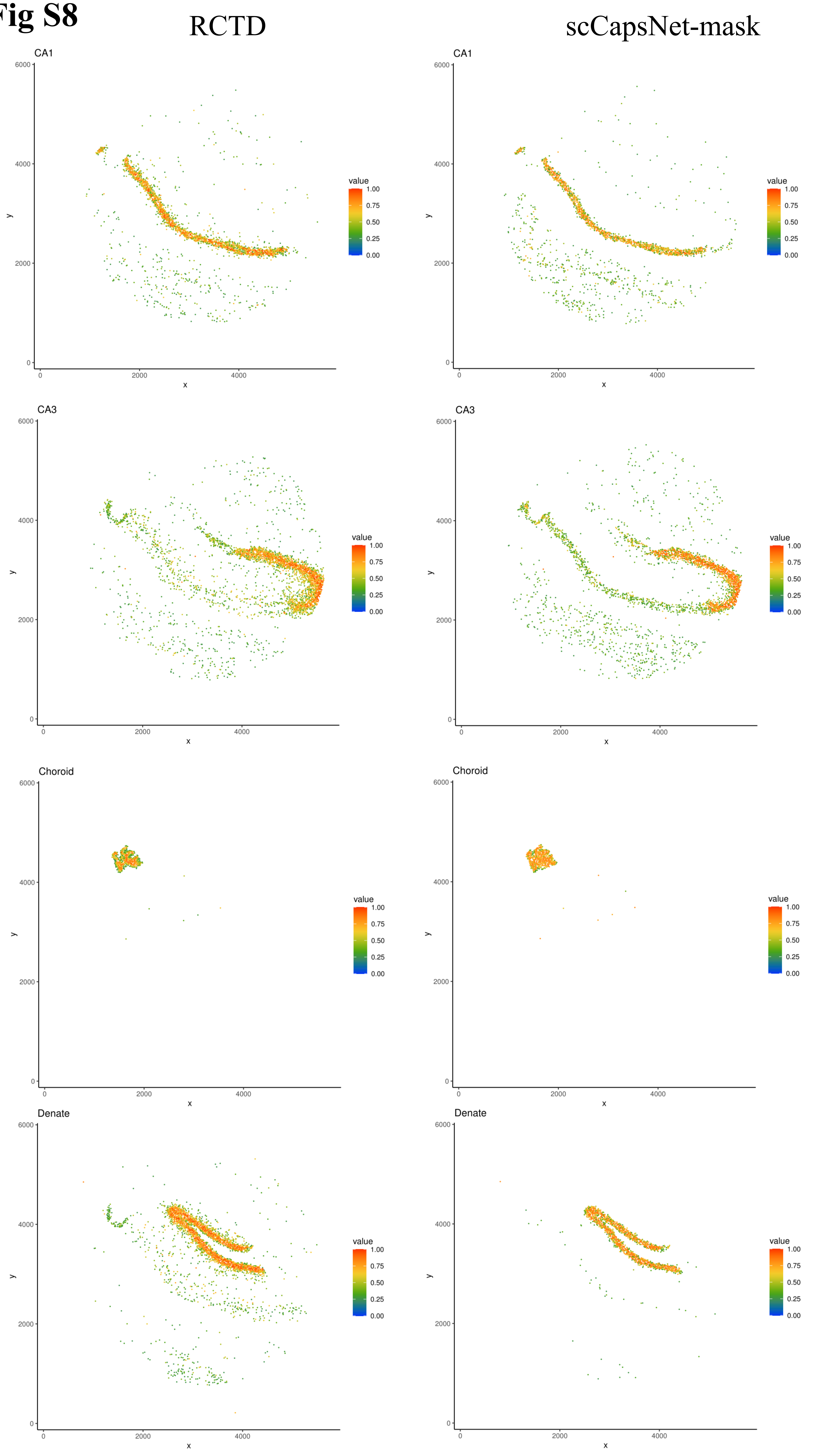
RCTD
scCapsNet-mask
CA1
CA3
Choroid
Denate
value
1.00
0.75
0.50
0.25
0.00
x
y
0
2000
4000
6000

Fig S9

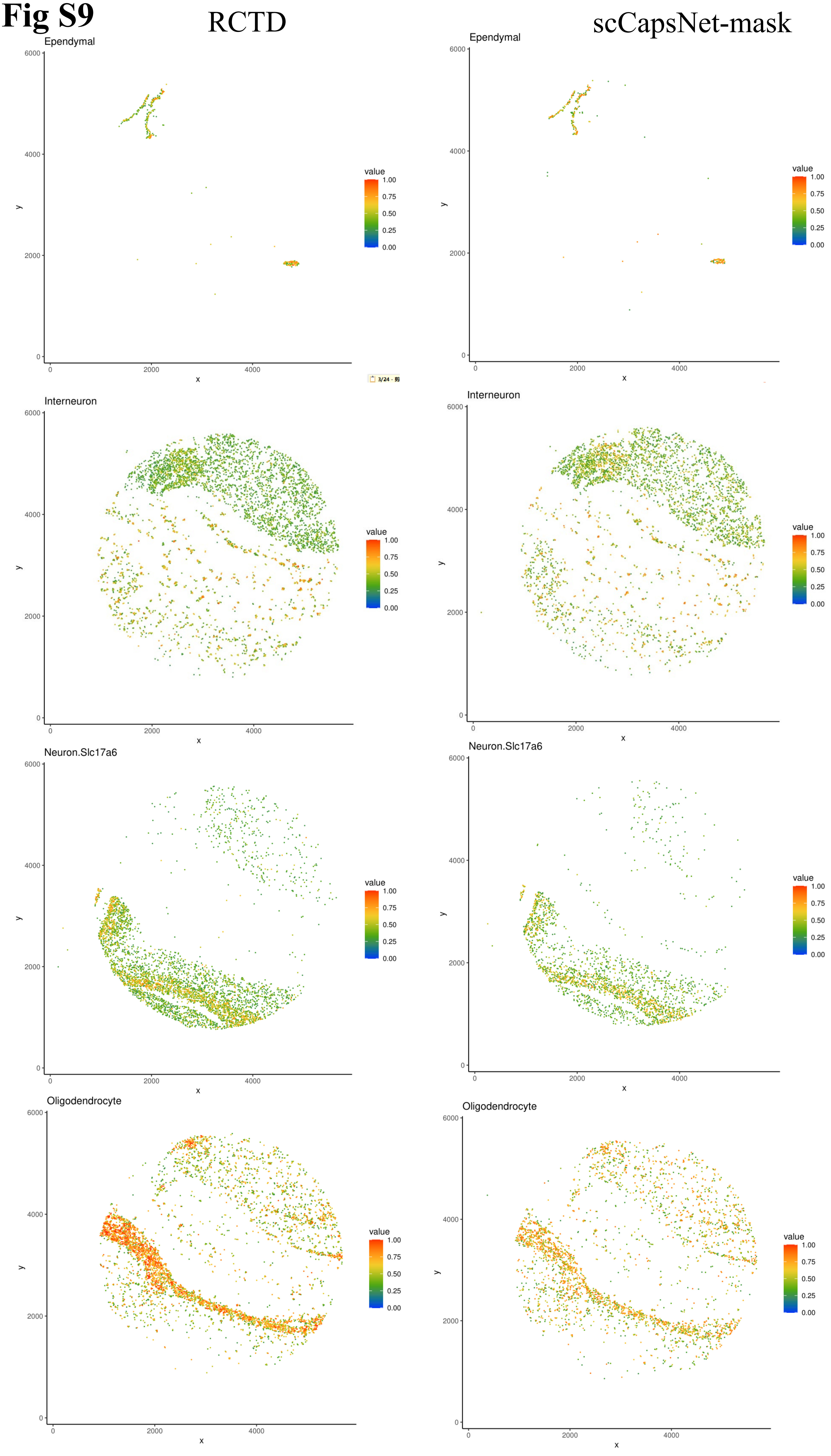

RCTD
scCapsNet-mask
Ependymal
Interneuron
Neuron.Slc17a6
Oligodendrocyte
value
1.00
0.75
0.50
0.25
0.00
x
y
0
2000
4000
6000

Fig S10

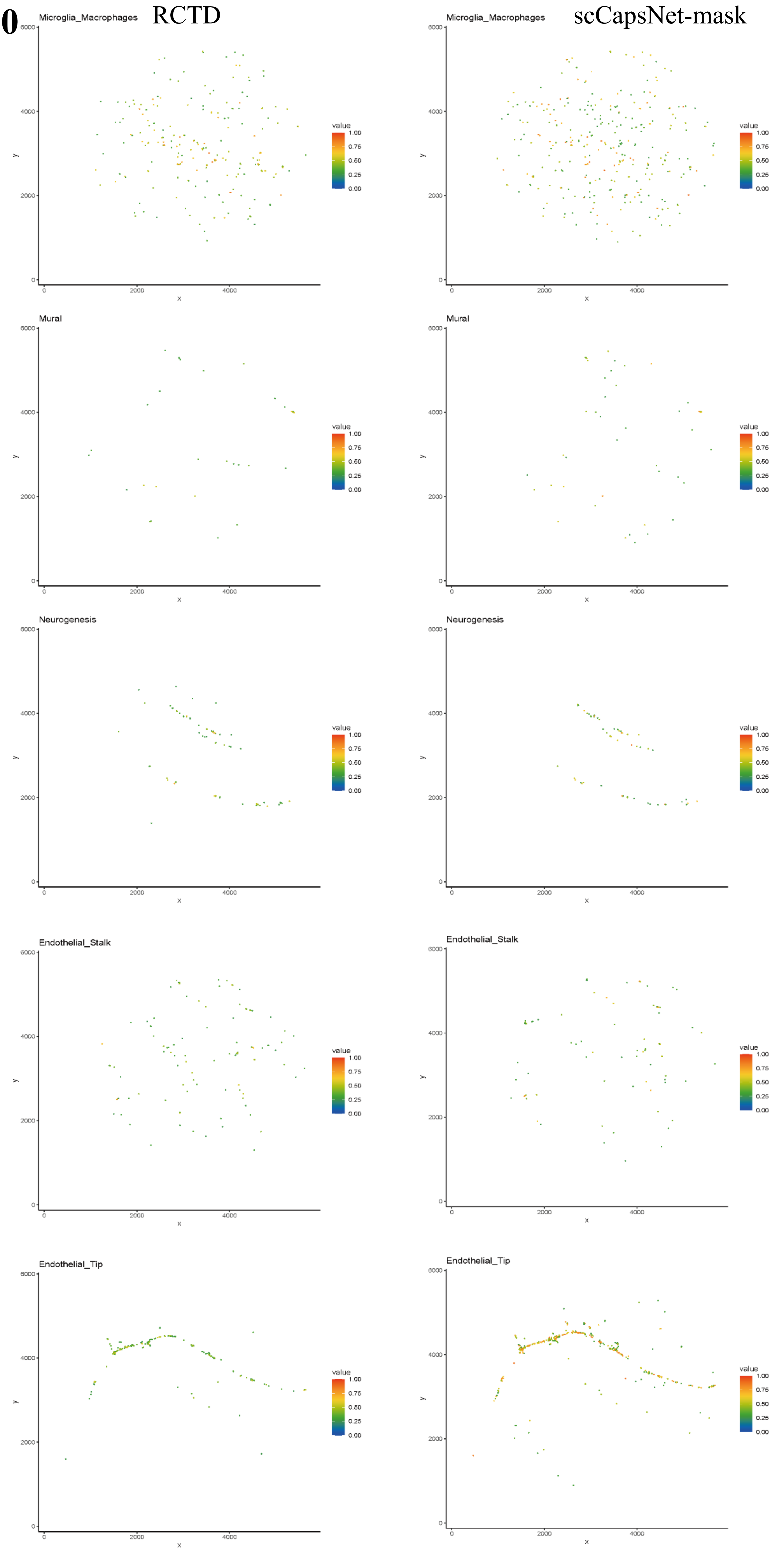
RCTD
scCapsNet-mask
Microglia_Macrophages
Mural
Neurogenesis
Endothelial_Stalk
Endothelial_Tip
value
1.00
0.75
0.50
0.25
0.00
x
y
0
2000
4000
6000

Fig S11

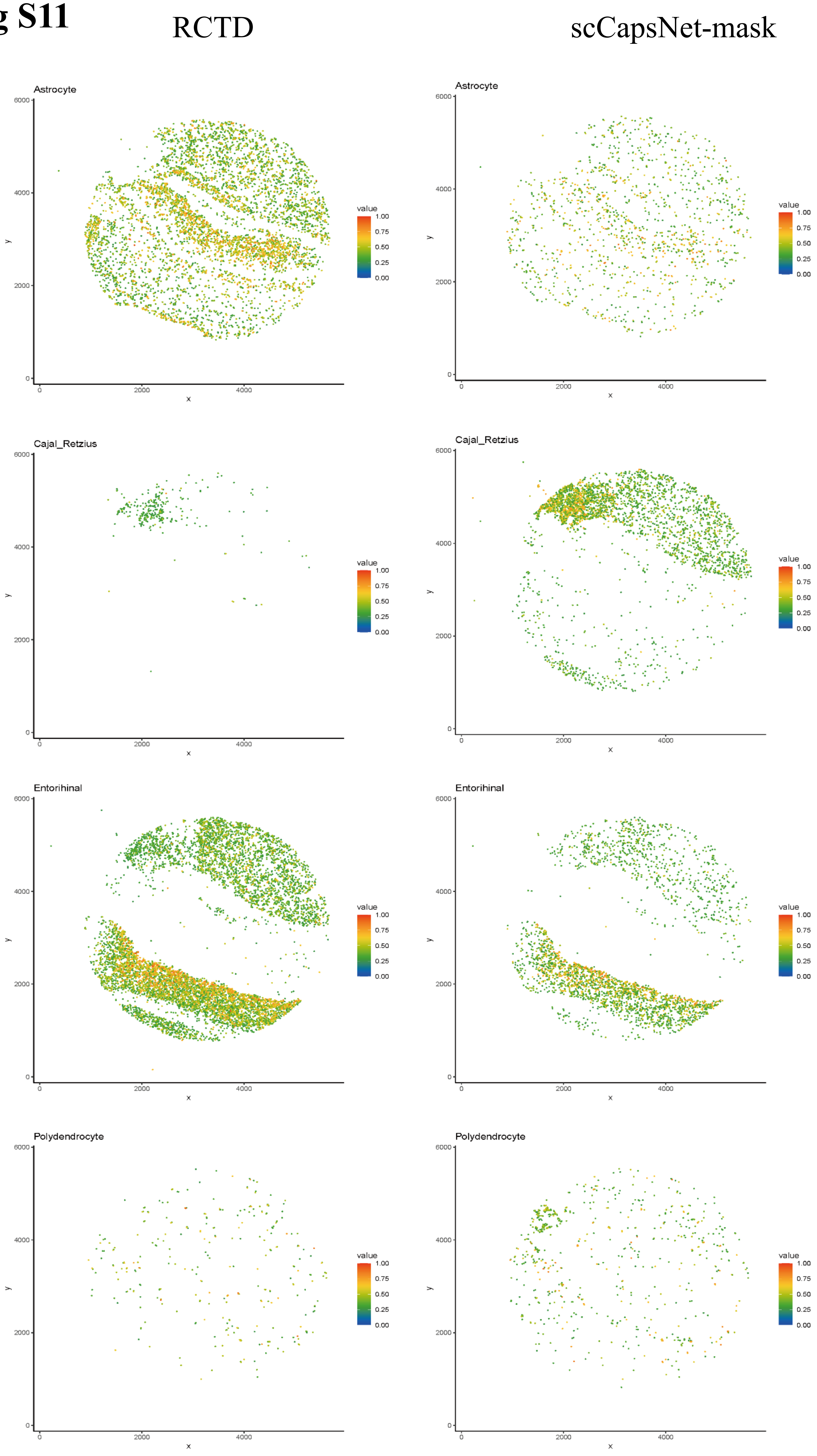
RCTD
scCapsNet-mask
Astrocyte
Cajal_Retzius
Entorihinal
Polydendrocyte
value
1.00
0.75
0.50
0.25
0.00
x
y
0
2000
4000
6000
